# 

**Published:** 1911-02

**Authors:** 


To My Subscribers.—I shall not be at Amarillo in May, and as it has been the custom of many to pay their subscription at the annual meeting of the Association I hereby request all who are in arrears to *please remit* on receipt of this number. Personal checks will be accepted. Bills were rendered last month. Please make it as easy with me as you can—to comply with the postal regulations, as I am required to drop delinquents.

February 1, 1911. F. E. Daniel.

